# Isolation of Antibacterial, Nitrosylmyoglobin Forming Lactic Acid Bacteria and Their Potential Use in Meat Processing

**DOI:** 10.3389/fmicb.2020.01315

**Published:** 2020-06-19

**Authors:** Yinglian Zhu, Qingli Yang

**Affiliations:** College of Food Science and Engineering, Qingdao Agricultural University, Qingdao, China

**Keywords:** lactic acid bacteria, nitrosylmyoglobin, antibacterial activity, meat, nitrite, colorant

## Abstract

The use of nitrite as a colorant and preservative in meat processing is associated with health risks. This study aimed to isolate nitrite-substituting lactic acid bacteria for use as natural biological colorants and preservatives. Among the 106 strains isolated from fermented foods, two strains with excellent ability to convert myoglobin and metmyoglobin (Met-Mb) to red nitrosylmyoglobin (Mb-NO) were selected. The superior ability to form Mb-NO was confirmed through UV-visible spectrophotometry, Fourier transform infrared spectrometry, electron spin resonance analysis, nitric oxide synthase activity assay, and Met-Mb reductase activity assay. The potent antibacterial activity was confirmed through biofilm and cytomembrane breakage of the indicator bacteria. Though performing 16S rDNA sequencing, they were identified as two different strains of *Lactobacillus plantarum*. Based on their favorable characteristics, their applications in the meat industry were further evaluated. This study identified a novel dual-function natural biological colorant and preservative to substitute nitrite in meat products. The application of the two strains would decrease the hazardous of nitrite to health.

## Introduction

Color is a very important sensory property for meat processing since it affects the freshness and quality of meat and meat products (Ramanathan et al., 2011; Biswas et al., 2012). The most common approach to retain color and freshness of meat products is to add food additives like nitrite to the meat products. Addition of nitrite can cure the red color of meat products and extend their shelf life. Unfortunately, the use of nitrite as a food additive can lead to the formation of strong carcinogens, resulting in the development of esophageal and stomach cancers on long-term consumption (Rosato et al., 2019). Consequently, the demand for nitrite-free meat products has increased, and the effort to develop nitrite substitutes for the curing of meat products has intensified.

Lactic acid bacteria (LAB) are a group of gram positive, non-spore forming, micro-aerophilic cocci and rods, which produce lactic acid (Domingos-Lopes et al., 2017). Since previous studies (Arihara et al., 1993) have reported the ability of *Lactobacillus fermentum* JCM1173 to convert brown metmyoglobin (Met-Mb) to bright red myoglobin derivatives, numerous researchers have demonstrated the chromogenic ability of LAB in meat products such as fresh meat, dry meat, etc. (Luo et al., 2013; Chen et al., 2016; Deraz and Khalil, 2018). The characteristic pink color of nitrite-free sausages can be achieved by using 10^8^ CFU/g of *L. fermentum* AS1.1880 as starting culture in meat batters (Zhang et al., 2007). Studies have also reported the beneficial effect of *Lactobacillus salivarius* on the color stability of fresh pork (Luo et al., 2013). The nitrite reductase activity of the strains promotes nitrozation of myoglobin and intensifies the formation of nitrosylmyoglobin (Mb-NO), which provides a typical pink color to meat products (Chen et al., 2016). Furthermore, the role of Nitric Oxide (NO) was studied while substituting nitrite with three lactic acid bacterial strains. The results showed that both nitrate reductase and nitric oxide synthase (NOS) participated in the formation of Mb-NO (Gou et al., 2019). All the aforementioned studies have demonstrated the great potential of LAB to form a characteristic pink color and maintain the color stability of meat products.

Additionally, LAB are considered as generally regarded as safe (GRAS) strains for human consumption (Liu et al., 2011). They are not only beneficial for the balance of the intestinal flora, but they also inhibit the growth of undesired microorganisms. Previous studies have shown that LAB inhibits the growth of spoilage organisms (Angmo et al., 2016) and fungal pathogens (Gerez et al., 2010). Thus, the addition of LAB strains could prolong the shelf-life of meat products. Moreover, the addition of LAB could result in large differences in organoleptic, biochemical, and flavor characteristics of food products, owing to their physiological features like substrate utilization, metabolic capabilities, and probiotic properties (Bao et al., 2012). Therefore, the selected LAB strains can function as ideal alternatives for nitrite, owing to their ability to intensify color and inhibit the growth of spoilage organisms. However, previously reported LAB didn’t have simultaneous antibacterial and Mb-NO-synthesizing functions, and NOS and Met-Mb reductase activity were low.

The objective of this study was to isolate bacterial strains with simultaneous Mb-NO-synthesizing and antibacterial functions for use as biological colorants and preservatives, thereby allowing for the substitution of nitrite, either partly or wholly, in meat products. The physiological characteristics of the isolates support their potential for use in the meat industry, like dynamic growth, acid production ability, and tolerance to chemical additives like NaCl and NaNO_2_, were assessed.

## Materials and Methods

### Strains

*Escherichia coli* (*E. coli*) (CGMCC 1.8723) and *Staphylococcus aureus* (*S. aureus*) (CGMCC 1.8721) were provided by the China General Microbiological Culture Collection Center (Beijing, China) and preserved in the Microbial Fermentation Engineering Laboratory, Qingdao Agricultural University (Qingdao, Shandong, China). Luria-Bertani (LB) medium was used for activation and culturing of the indicator bacteria.

### Isolation of LAB Strains From Fermented Products

The isolation of LAB was performed in accordance with a previously reported protocol (Luo et al., 2013). Sausage samples (25 g) were cut into pieces and added to 225 mL of sterile saline solution (0.85%). The mixture was homogenized for 90 s to obtain a 1:10 sample homogenate with a BagMixer (400CC, Interscience, Saint Nom, France).

For yogurt, 25 mL of yogurt sample was added to 225 mL of sterile saline solution (0.85%) containing glass beads, and the mixture was shaken thoroughly to obtain a 1:10 sample homogenate. The samples continued to be diluted to 10-fold series. Subsequently, 0.1 mL of this dilution was spread on an MRS agar plate containing 2% CaCO_3_ and incubated at 37°C for 48 h. The gram positive colonies, identified by the presence of a clear dissolved calcium circle surrounding the colony on the agar plate, were picked as candidate LAB strains.

### Chromogenic Effect in Met-Mb-Containing Medium

Met-Mb solution (20 mg/mL) was heated at 50°C for half an hour to inhibit any residual Met-Mb reductase activity. Following filtration on a microporous membrane, Met-Mb was added to MRS medium to attain a final myoglobin concentration of 2 mg/mL. The selected strains were inoculated, and immediately covered with a layer of paraffin oil to prevent oxidation during incubation. Subsequently, the strains were incubated anaerobically at 37°C until the medium turned red. The strains were not inoculated in the control group.

### Antibacterial Activity

The strains were further screened for their antibacterial activity. The selected strains were cultivated at 37°C for 24 h, and centrifuged at 10,000 × *g* for 10 min, at 4°C. The supernatant pH was adjusted with NaOH (1 mol/L) to 6.0 to eliminate the interference of organic acids, and then was used to test for antibacterial activity against *E. coli* and *S. aureus* through agar well diffusion method in LB agar plate with the diameter of Oxford cup 6 cm (Gerez et al., 2010).

### Physiological and Biochemical Reactions

The selected strains were studied for certain physiological and biochemical reactions consisting of amino acid decarboxylase activity (including lysine, ornithine, and arginine), glucose aerogenesis, lipoxidase activity, and their ability to produce lactic acid and H_2_S (Luo et al., 2013).

### Chromogenic Effect in Meat Products

Fresh lean pork was purchased from the local supermarket and minced. The control contained 3% sodium chloride, 2% glucose, and 0.01% nitrite. The inoculated groups (nitrite-free) contained 3% sodium chloride, 2% glucose, and the appropriate volume of inoculum (about 8 log CFU/g of batters). Each sample was enclosed in plastic intestines with 100 g of batters and cured at 4°C for 24 h, then fermented at 37°C for 4 h in 85% humidity. The sausages were baked in a smoke house at 85°C for 1.5 h until the internal temperature reached 74°C. Subsequently, they were smoked at 85°C for 3.5 h using wood chip, and cooled below 10°C for storage. The chromatic value of the meat samples was evaluated using the CIELAB system (CromaMeter CR-400, Conica Minolta, Japan), and the average value of lightness (*L*^∗^), redness (*a*^∗^), and yellowness (*b*^∗^), was recorded (Cullere et al., 2018).

### Effect of Mb-NO Formation

The selected strains were cultured at 37°C anaerobically until the inoculum turned red. Subsequently, the inoculum was centrifuged at 10,000 × *g* for 10 min, at 4°C. The Mb-NO formed was detected by measuring the absorption of the supernatant using a UV-Vis spectrophotometer (UV-6000PC, Shanghai Metash Instruments Co., Ltd., China) at wavelengths ranging from 400 to 700 nm, at 1 nm intervals, in accordance with a previously reported protocol (Møller et al., 2003). The presence of Mb-NO in the supernatant was further determined by the electron spin resonance (ESR) analysis method described previously (Gøtterup et al., 2007), with minor alternations. Each sample (0.2 mL) was transferred to an ESR tube and analyzed on a Bruker ESC 106 spectrometer (JES-TE2X, JEOL Ltd., Tokyo) using the following conditions: microwave power, 4 mW; modulation frequency and width, 100 kHz and 1.0 mT; temperature, 77 K; measurement time, 8 min.

Extraction of NO-Mb from meat products was performed in accordance with a previously reported protocol (Gao et al., 2014), with slight modifications. The fermented samples (10 g) were minced and homogenized in 90 mL phosphate buffer (pH 6.0, 20 mM) for 1.5 min with a high-speed beating BagMixer (400CC, Interscience, Saint Nom, France). Following incubation in a dark room at 4°C for 1 h, the homogenates were centrifuged at 6000 × *g* for 10 min. The extracted, cured pigment was filtered on a nitrocellulose membrane. The Mb-NO analysis was performed using a UV-Vis spectrophotometer (UV-6000PC, Shanghai Metash Instruments Co., Ltd., China) at wavelengths ranging from 350 to 700 nm, at 1 nm increments. The Fourier transform infrared (FTIR) spectra of the extracted pigment was analyzed from 550 to 3750 cm^–1^ on a FTIR Spectrometer (Nexus 470, Nicolet Instrument Corp, United States).

### NOS Activity and Met-Mb Reductase Activity

Enzyme extraction was performed following cultivation of the strains at 37°C for 24 h. After centrifugation at 10,000 × *g* for 10 min, at 4°C, the cell pellets were washed thrice using 2.0 mM phosphate buffer solution (PBS, pH 7.0) and the supernatant was stored as later use. The washed cell pellets were re-suspended in 4 mL of 2.0 mM PBS (pH 7.0), and subjected to ultrasonic disruption at 4°C for 10 min, with 800 W power and 50% amplitude. Following disruption, the cell supernatant was mixed with the previously stored supernatant and used as enzyme extracts to detect the enzyme activity of the cells.

NOS activity was measured according to the method described by Luo et al. (2013). The NOS assay reaction system (1 mL) comprised of 50 mM PBS (pH 7.0), 1.0 mM CaCl_2_, 10 μM Flavin Adenine Dinucleotide (FAD), 10 μM Flavin Mononucleotide (FMN), 0.1 mM Nicotinamide Adenine Dinucleotide Phosphate (NADPH), and 0.5 mL of enzyme extracts. The reaction was initiated by the addition of L-arginine (1.0 mM). NADPH was oxidized during the conversion of L-arginine to L-citrulline, and its consumption was assessed by the decrease in absorbance at 340 nm. NOS activity was determined as the NADPH consumption per min per ml of enzyme extract.

Met-Mb reductase activity was measured according to the method described by Luo et al. (2013). The assay mixture contained 0.1 mL of 5.0 mM EDTA, 0.1 mL of 50 mM PBS (pH 7.0), 0.1 mL of 3.0 mM K_4_Fe(CN)_6_, 0.1 mL of ultrapure water, 0.2 mL of 0.75 mM Met-Mb in 2.0 mM PBS (pH 7.0), 0.3 mL of enzyme extract, and 0.1 mL of 2.0 mM NADH. The reaction was initiated by the addition of NADH at 25°C, and the absorbance at 580 nm was measured every 12 s for 5 min, until no change in absorbance was observed. The difference in the absorbance at 580 nm for Met-Mb and Oxymyoglobin (MbO_2_) reached the maximum, and the molar extinction coefficient was 1.2 × 10^4^ L/molcm. Met-Mb reductase activity was defined as the change in absorbance per min per ml of enzyme extract.

### Antibacterial Characteristics of the Selected Strains

Dynamic growth of indicator bacteria: After culturing *E. coli* and *S. aureu*s at 37°C for 24 h and centrifugation at 10,000 × *g* for 10 min at 4°C, the cells were collected and suspended in LB broth to obtain a bacterial concentration of 10^5^ CFU/mL. Subsequently, 5 mL of the bacterial suspension was mixed with an equal volume of the fermentation supernatant of the selected strains and incubated at 37°C. Dynamic growth of the indicator bacteria was assessed every 2 h by plotting the absorbance curve at 600 nm according to the method of Lv et al. (2018), using a UV spectrophotometer (TU-1810, Purkinje, China). The LB broth which provided the substitute for the fermentation supernatant of the selected strains, was used as the control.

Fluorescence spectrum analysis: After being centrifuged at 10,000 × g for 10 min, at 4°C, and washed thrice using PBS (pH 7.0), the above treated indicator bacterial cells were harvested. Then the indicator bacterial cells were stained by addition of 20 μL fluorescein diacetate (FDA), 60 μL propidium iodide (PI), and 920 μL sterile saline solution (0.85%), and placed in the dark for 12 h at 4°C (Yu et al., 2017). A fluorescent spectrum scan was conducted using a fluorescence spectrophotometer (F-4600, HITACHI, Japan), at an excitation wavelength of 450 nm.

Fluorescence microscope analysis: The stained indicator bacterial cells were centrifuged at 10,000 × g for 10 min at 4°C and washed three times with PBS (pH 7.0), and then re-suspended in 1 mL PBS (pH 7.0). The re-suspended cells were detected using a fluorescence microscope.

### 16S rDNA Sequence Analysis

The selected strains were identified by 16S rDNA sequence analysis. The template DNA of each strain was extracted in accordance with a previously reported protocol (Minas et al., 2011). The PCR mix (50 μL) included template DNA (1 μL), forward primer (2 μL), reverse primer (2 μL), dNTPs (4 μL), Taq DNA polymerase (1 μL), PCR buffer (with Mg^2+^) (5 μL), and ddH_2_O (35 μL),. The 16S rDNA was amplified by PCR using the following universal primers: forward primer 8F (5′-AGAGTTTGATCCTGGCTCAG-3′), and reverse primer 1492R (5′-TACGGCTACCTTGTTACGACTT-3′). The PCR amplification program comprised of pre-denaturation at 94°C for 5 min, and 30 cycles of denaturation at 94°C for 30 s, annealing at 55°C for 30 s, and extension at 72°C for 1.5 min, followed by further extension at 72°C for 10 min with a PCR instrument (PCT-1148, BIO-RAD, United States). The PCR products were separated by agarose gel electrophoresis (1%, w/v) at 80 V for 30 min, and the gels were scanned using a gel documentation system, and analyzed using the DNA Bio Imaging Systems Software. The 16S rDNA sequencing was performed at Sangon Biotechnology Co., Ltd. (Shanghai, China). The sequences were acquired using the BioEdit software, and submitted to the National Center for Biotechnology Information (NCBI), and their accession numbers were MH357373 and MH357374, respectively. Thereafter, the sequences were compared with the sequences in the GenBank database using the Basic Local Alignment Search Tool (BLAST) program^1^.

### Physiological Characteristics of the Isolates for Meat Processing Use

Following cultivation of the selected strains at 37°C for 24 h, 1 mL of the inoculum was added to 100 mL of MRS broth and incubated at 37°C. Dynamic growth of each strain was assessed by measuring the absorbance at 600 nm, every 2 h using a UV spectrophotometer (TU-1810, Purkinje, China), as described by Polak-Berecka et al. (2013), with minor modifications. The tolerance of the selected strains to sodium chloride was examined by inoculating the strains in MRS broth containing 0 (control), 2, 4, 6, 8, 10, and 12% (w/v) NaCl, respectively, and incubating at 37°C for 24 h. The tolerance of the selected strains to sodium nitrite was detected by inoculating the strains in MRS broth containing 0 (control), 20, 40, 60, 80, 100, and 120 mg/mL sodium nitrite, respectively, and incubating at 37°C for 24 h. The cell density of each strain following cultivation was assessed by measuring the absorbance at 600 nm, using a UV spectrophotometer (TU-1810, Purkinje, China). The acid production ability of the selected strains was examined by inoculating the strains in MRS broth, and incubating at 37°C. The pH of the broth was measured using a pH meter (ORP-013M, Kelilong Electron Co., Ltd., China) at 0, 2, 4, 6, 8, 10, and 12 h, respectively.

### Statistical Analysis

Statistical analysis was carried out with SPSS Version 18.0 and the data was analyzed with multiple comparisons with Duncan method. Probability level of 0.05 was accepted as a significance limit.

## Results

### Isolation of LAB Strains

One hundred and six Gram positive strains were selected in terms of colony morphology, size, presence of a calcium dissolved circle, and Gram staining. Among them, 12 strains with chromogenic effect were picked and inoculated in Met-Mb-containing medium. The antibacterial activity of the 12 strains is listed in Table 1. Table 1 shows that strains e, 2, and 33 had the best antibacterial activity against *S. aureus*, while strains b, d, e, 2, 32, and 33 had the best antibacterial activity against *E. coli.* Consequently, the six strains b, d, e, 2, 32, and 33 with the value (D2/D1 and D3/D1) over 2.0 were selected for further study.

**TABLE 1:** Antibacterial activity of the two selected strains.

Strains	*E. coli* (D2/D1)	*S. aureus* (D3/D1)
Strain a	1.81 ± 0.12^a^	1.64 ± 0.05^a^
Strain b	1.94 ± 0.22^abcde^	1.88 ± 0.21^abc^
Strain c	1.75 ± 0.06^a^	1.86 ± 0.11^ab^
Strain d	2.22 ± 0.07^e^	1.88 ± 0.08^abc^
Strain e	2.18 ± 0.11^de^	2.21 ± 0.15^d^
Strain 2	2.14 ± 0.10^bcde^	2.11 ± 0.09^bcd^
Strain 3	1.85 ± 0.09^ab^	1.71 ± 0.10^a^
Strain 8	1.88 ± 0.21^abc^	1.73 ± 0.28^a^
Strain 18	1.73 ± 0.33^a^	1.85 ± 0.15^ab^
Strain 32	2.03 ± 0.08^abcde^	1.90 ± 0.08^abc^
Strain 33	2.16 ± 0.10^cde^	2.15 ± 0.02^cd^
Strain 44	1.93 ± 0.22^abcd^	1.65 ± 0.19^a^

*Values are represented as means ± SD from triplicate experiments. different lowercase superscript in the same column indicates that the antibacterial effect is significantly different (P < 0.05); D2 denotes the diameter of the zone of inhibition against E. coli; D3 denotes the diameter of the zone of inhibition against S. aureus; D1 denotes the diameter of Oxford cup; The ratio of diameter (D2/D1 and D3/D1) is used to indicate antibacterial activity.*

The physiological and biochemical characteristics of the six strains are listed in Table 2. The results showed that all six strains produced lactic acid, did not show any H_2_S producing capability, lipoxidase activity, and lysine, ornithine, arginine decarboxylase activity. However, strain b and strain 33 had glucose aerogenesis capability, which might have negative effect on the texture of meat products. Consequently, the four strains d, e, 2, and 32 were chosen for further chromogenic activity study.

**TABLE 2:** Physiological and biochemical characteristics of the six strains.

Strain	Lactic acid production	H_2_S production	Lysine decarboxylase activity	Ornithine decarboxylase activity	Arginine decarboxyase activity	Glucose aerogenesis	Lipoxidase activity
Strain b	+	−	−	−	−	+	−
Strain d	+	−	−	−	−	−	−
Strain e	+	−	−	−	−	−	−
Strain 2	+	−	−	−	−	−	−
Strain 32	+	−	−	−	−	−	−
Strain 33	+	−	−	−	−	+	−

*The sign “+” denotes a positive reaction, and “−” denotes a negative reaction.*

### Chromogenic Activity in Meat Products

**FIGURE 1:**
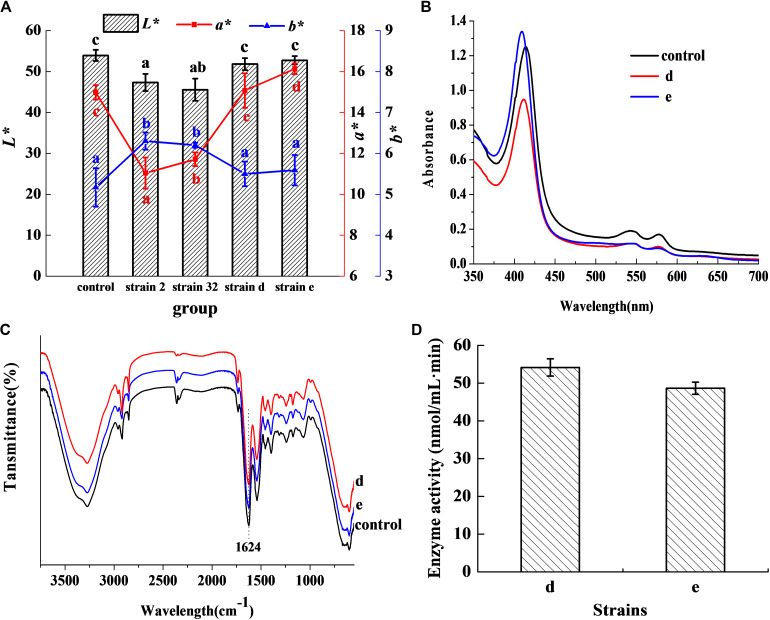
Chromogenic activity in meat products. Chromogenic activity of the selected strains in meat products **(A)**. Histogram denotes the value of L* and line chart denotes the value of a* and b*. Visible absorption spectra of pigment extracted from cured meat products **(B)**. FTIR spectra of pigment extracted from 3750 to 550 cm^–1^ **(C)**. NOS activity of the two selected strains **(D)**.

In Figure 1A, the *L*^∗^ value of the inoculated groups d and e was 51.82 and 52.75, respectively, which showed no significant difference in comparison with the control (*P* > 0.05). The *a*^∗^ value was 15.07 for group d, which was no significant difference with the control (*P >* 0.05), but higher than other inoculated groups (*P* < 0.05). The *a*^∗^ value of group e was 16.13 and was higher than both the control and other inoculated groups (*P* < 0.05). The *b*^∗^ value of the groups d and e was lower than other inoculated groups (*P* < 0.05), and had no significant difference in comparison with the control (*P* > 0.05). The results indicated that the strains d and e had excellent chromogenic activity in meat products, as compared to the control and other strains. Figure 1B shows the absorbance peaks of the two inoculated groups d and e, and the control, which were at 421, 548, and 579 nm, respectively. These values almost matched the typical absorbance peaks of Mb-NO (Gao et al., 2014). In the FTIR of extracted pigment, the bands at 1624 cm^–1^ of the three groups (Figure 1C) coincided with the stretching frequency of Fe-NO (Maxwell and Caughey, 1976), which further confirmed that Mb-NO was present in the control and the two inoculated groups, d and e. Figure 1D indicated that both the selected strains d and e had NOS activity.

### Mb-NO Formation Effect

**FIGURE 2:**
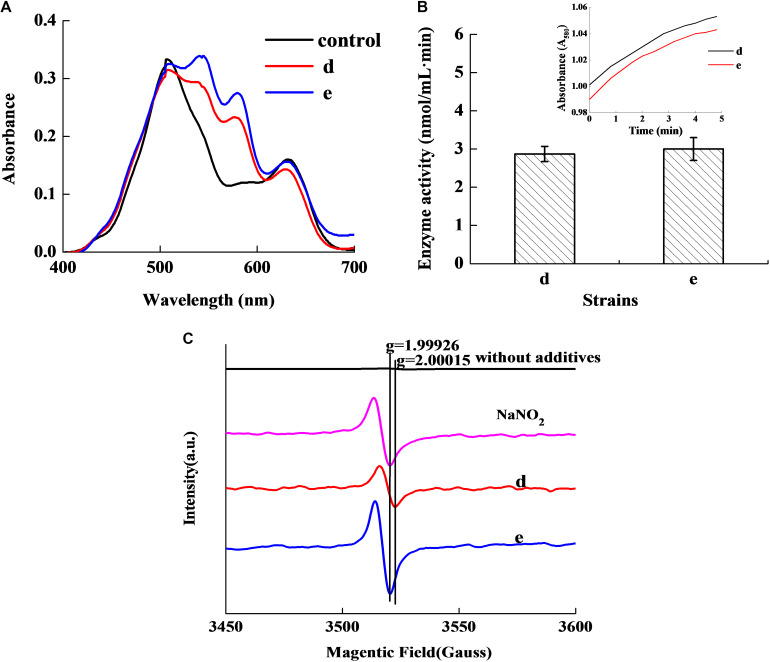
Chromogenic activity of the two selected stains in Met-Mb-containing medium. Visible absorption spectra of pigment extracted from Met-Mb-containing MRS inoculum **(A)**. Met-Mb reductase activity of the two selected strains **(B)**. ESR spectra of pigment extracted from Met-Mb-containing MRS inoculum **(C)**.

Figure 2A revealed that the control group had two absorbance peaks at approximate wavelengths of 505 and 635 nm, which are the typical absorbance peaks of Met-Mb (Li et al., 2016). However, both experimental samples had two new absorbance peaks at approximate wavelengths of 545 and 579 nm, which were consistent with the typical absorbance peaks of Mb-NO (Gao et al., 2014). Figure 2B shows that both the selected strains d and e possessed Met-Mb reductase activity. Met-Mb reductase activity was calculated as the Met-Mb reduced during the initial linear phase when the absorbance values at 580 nm increased in a short time. The presence of Mb-NO was further verified by ESR as seen in Figure 2C. Significant ESR signals of the g factors (around 2.0) were observed in groups with NaNO_2_ addition, strains d and e inoculation, while no signal of the g factors (around 2.0) was detected in the control group lacking any additives.

### Antibacterial Ability

**FIGURE 3:**
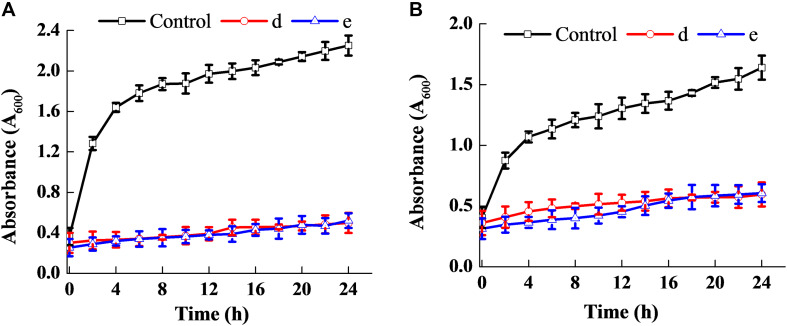
Antibacterial ability of the two selected strains. Effect on the dynamic growth of *S. aureus* **(A)**. Effect on the dynamic growth of *E. coli* **(B)**.

**FIGURE 4:**
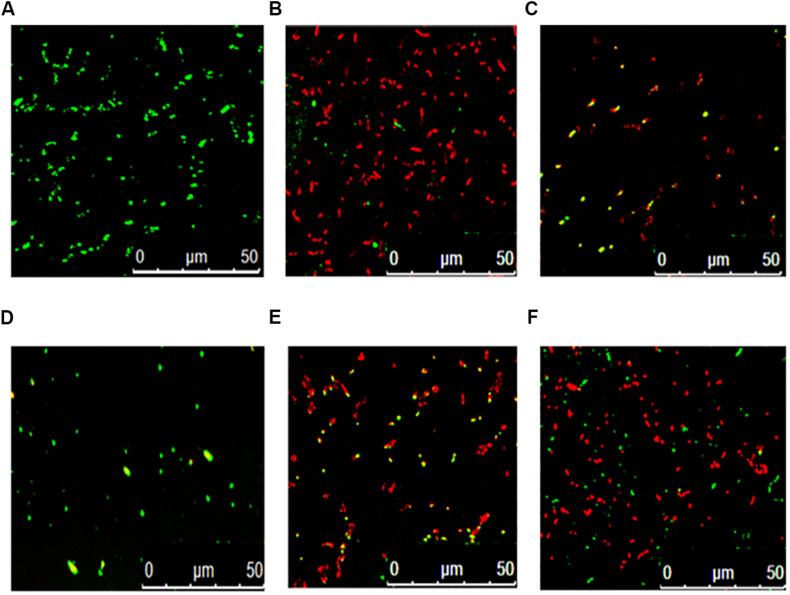
Fluorescence microscopic spectrum of the two indicator bacteria dyed with FDA and PI: *E. coli* as control **(A)**, *E. coli* treated with fermentation supernatant of strain d **(B)**, *E. coli* treated with the fermentation supernatant of strain e **(C)**, *S. aureus* as control **(D)**, *S. aureus* treated with the fermentation supernatant of strain d **(E)**, *S. aureus* treated with the fermentation supernatant of strain e **(F)**.

**FIGURE 5:**
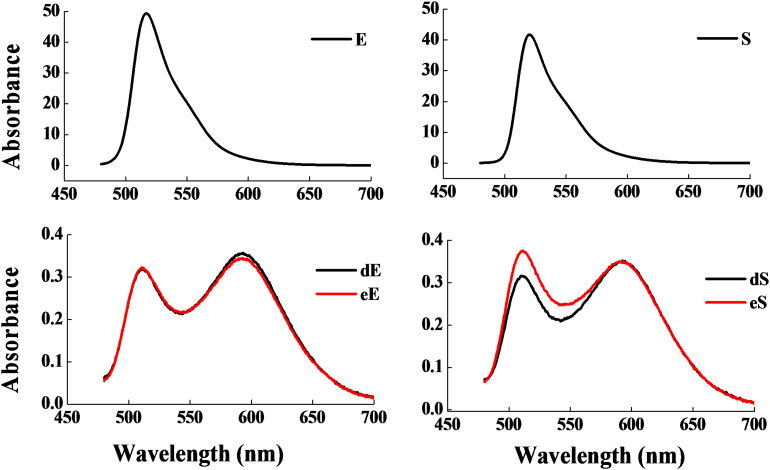
Fluorescence scan spectrum of the two indicator bacteria dyed with FDA and PI: *E. coli* as control (E), *E. coli* treated with the fermentation supernatant of strains d (dE), *E. coli* treated with the fermentation supernatant of strain e (eE), *S. aureus* as control (S), *S. aureus* treated with the fermentation supernatant of strain d (dS), *S. aureus* treated with the fermentation supernatant of strain e (eS).

Figure 3 shows that the fermentation products of the selected strains, d and e, had the ability to significantly inhibit the dynamic growth of the two pathogenic bacteria, *S. aureus* and *E. coli*, with negligible change in the total counts of the two bacteria. Figure 4 shows the fluorescence microscopic spectrum of the two indicator bacteria dyed with FDA and PI. Yellow-green fluorescence was observed in the control and red fluorescence mostly appeared in the two experimental samples. Fluorescence scan spectrum revealed that the untreated indicator bacteria had only one peak at 518 nm, which was the absorbance peak of FDA (Figure 5). However, a new absorbance peak appeared at 597 nm following treatment of the bacteria with the fermentation products of strains d and e.

### Results of 16s rDNA

**FIGURE 6:**
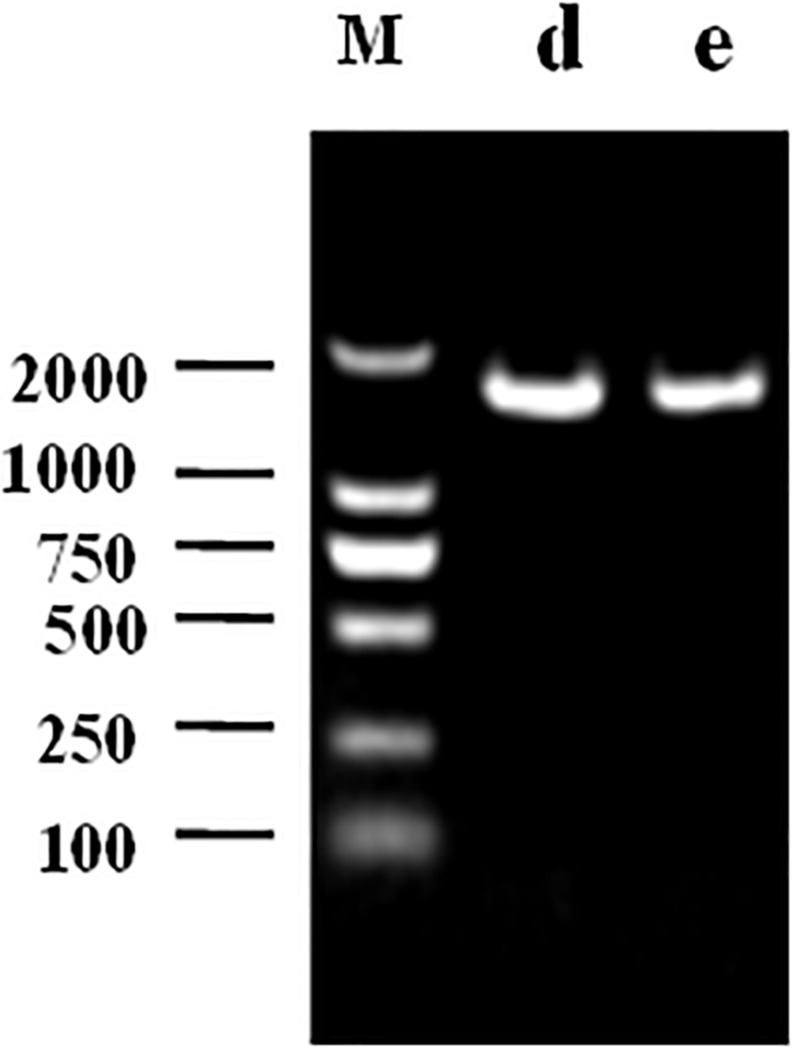
Agarose gel electrophoresis following PCR amplification: M denotes DNA Marker, d denotes strain d, e denotes strain e.

**FIGURE 7:**
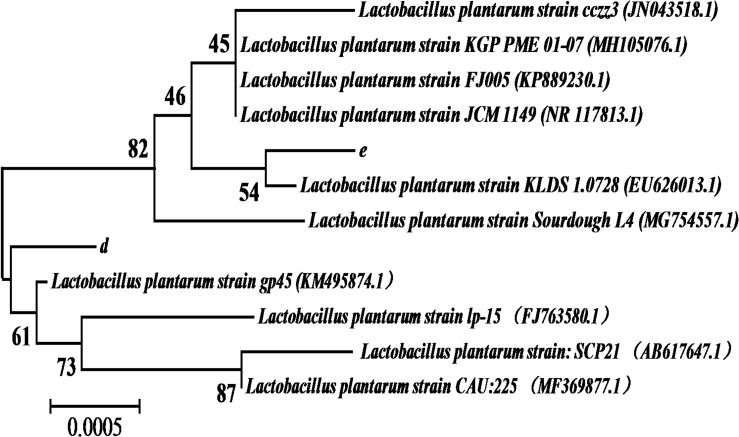
The 16S rDNA sequence alignment of strains d and e.

The 1468 and 1470 bp 16s rDNA fragments of strains d and e obtained during agarose gel electrophoresis are shown in Figure 6. BLAST analysis of the base sequence of 16s rDNA revealed that the two strains d and e were homogenous to *L. plantarum* (Figure 7), and the similarity was 99%. The sequences have been deposited in the GenBank database with the accession numbers MH357373 and MH357374, respectively. The strains have been stored in the Microbial Fermentation Engineering Laboratory, Qingdao Agricultural University, China, and China General Microbiological Culture Collection Center, and the strain numbers are CGMCC 17078 and CGMCC16130, respectively.

### Physiological Characteristics of the Isolates for Meat Processing Use

Figure 9A shows that the lag phase of the two strains were short, and the logarithmic phase of both strains began at the fourth hour, owing to their fast growth rate. Figure 9B reveals that a sodium chloride concentration less than 4% had no effect on the growth of the two strains, and that they were able to grow relatively well at a sodium chloride concentration of 10%. Figure 9C revealed that the strains had good sodium nitrite tolerance. On addition of 120 mg/mL of sodium nitrite, the absorbance values of the inoculum reached above 0.6. Furthermore, as seen in Figure 9D, the two strains rapidly reduced the pH of the fermentation broth. Therefore, the physiological characteristics of the isolates illustrated that they had good potential for use in the meat industry.

## Discussion

As demonstrated, the typical absorbance peaks of oxymyoglobin (MbO) were at 544 and 582 nm, while those of Mb-NO were at 548 and 579 nm, respectively (Millar et al., 1996). The typical peaks of Mb-NO were detected in the experimental samples as well as the control (Figure 1B), which indicates that the two strains in meat products produce chromogenic effects similar to that of nitrite. Additionally, previous research has reported that the stretching frequency of Fe-NO in FTIR was in the range of 1600–1700 cm^–1^ (Maxwell and Caughey, 1976). Hence, the bands at 1624 cm^–1^ (Figure 1C) correspond to the stretching frequency of Fe-NO, which further confirms that the two strains form Mb-NO in a nitrite-free manner. Mb-NO is an important pigment responsible for the attractive pink color of meat products, and it forms a stable color on heating (Kim et al., 2019). Mb-NO in the control sample was generated through the interaction between myoglobin and NO, which was produced from nitrite (Gao et al., 2014). Therefore, the two strains inoculated in meat products generated NO during the fermentation process, which was further confirmed by presence of NOS (Figure 1D). So the NOS pathway is the major source of NO in the two strains, which forms Mb-NO instead of depending on nitrite, and the NOS function was also proposed in strains *Lactobacillus salivarius* (Luo et al., 2013) and coagulase-negative *staphylococci* (Huang et al., 2019). But the NOS activity of the two strains (54.15 and 48.65 nmoL/mLmin) was significantly higher than that previously reported (34.14 nmoL/mLmin) (Luo et al., 2013), which constitutes a novel finding herein, showing that the two strains had robust Mb-NO-synthesizing and nitrite-substituting potential.

The absorption bands of the experimental samples (Figure 2A) indicated that the two strains can revert Met-Mb, which also confirmed by Met-Mb reductase activity of the strains (Figure 2B). The presence of Met-Mb might cause the meat to appear brown. The Met-Mb reductase was the controlling factor in retarding the accumulation of Met-Mb (Chiou et al., 2001) and played key role in the color stability of meat products (Djimsa et al., 2017). As shown in Figure 2C, the ESR signals of the g factors (around 2.0) in the experimental samples and the control were all detected, which showed paramagnetic characteristics of typical penta-coordinate Mb-NO (Gøtterup et al., 2007). Thus, ESR result indicates the existence of Mb-NO as one of the Met-Mb reduction products. The Met-Mb reductase activity of strains d (2.87 nmoL/mLmin) and e (3.01 nmoL/mLmin) was higher than that reported previously (0.21 nmoL/mLmin), indicating that the two strains can regulate color stability in meat products than previously reported strains by the reduction of Met-Mb greatly.

FDA emitted yellow-green fluorescence under a blue laser when it was bound to living cells (Xiao et al., 2011). However, PI was a cell-membrane impermeable fluorescent dye which exclusively combined with DNA in cells that were dead or had broken membranes and emitted red fluorescence (Boyd et al., 2008). Fluorescence microscopic analysis illustrated that the fermentation products of strains d and e destroyed the biofilm and cytomembrane of indicator bacteria, resulting in most cell death (Figures 4B,C,E,F). For fluorescence spectrum, compared with the control, the new absorbance peaks were typical absorbance peaks of PI (Figure 5), which also indicated the destruction of biofilm and cytomembrane of the indicator bacteria by the fermentation products of strains d and e. Peaks at 518 nm had a blue-shift, which attributed to the fewer amount of the treated bacteria compared to the control. The stronger antibacterial ability of the two strains attributed to the Lacidophilin production during fermentation (Liu et al., 2017), which damage the protective effect of biofilm and cytomembrane of the indicator bacteria. Several of the *L. plantarum* strains have been used as probiotics (Geeta and Yadav, 2017). Hence, they had potential to replace nitrite as preservative in meat products. This is an innovative result for natural biological colorants that has not confirmed by other studies.

**FIGURE 8:**
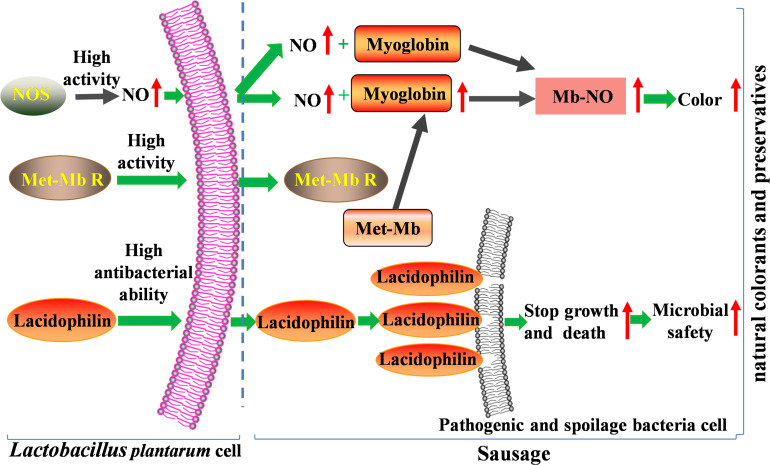
Mechanism of the two isolated strains improving color and microbial safety used as natural colorants and preservatives in meat products.

Therefore, in the present study, high activity of NOS and Met-Mb reductase of the two strains promote accumulation of nitric oxide and myoglobin, which result in the Nb-NO production and color formation increase significantly. Moreover, high antibacterial ability of lacidophilin induces most pathogenic and spoilage bacteria to death through cell-membrane destruction, which enhances microbial safety of meat products. So the two isolated strains with superior dual functions have great potential to substitute nitrite (Figure 8).

**FIGURE 9:**
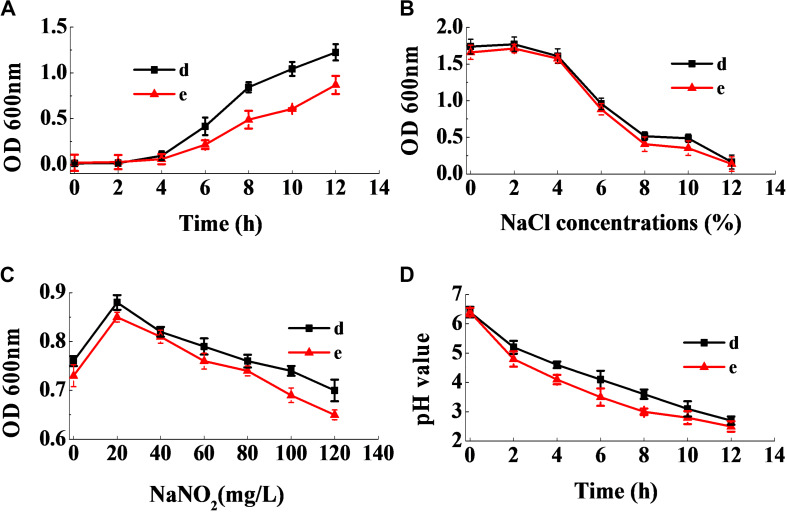
Dynamic growth curve of the two selected strains **(A)**. The resistance of the two selected strains to NaCl **(B)** and NaNO_2_ **(C)**, and their acid-producing ability **(D)**.

The physiological, biochemical and phenotypic characteristics are the basis for identifying LAB, and also the prerequisite for exploring its potential value (Guley et al., 2015; Vasiee et al., 2018). The amino acid decarboxylase activity has been described in many microorganisms. However, lysine, ornithine and arginine decarboxylase, leads to the production of cadaverine, putrescine, and spermine, respectively, which increases the toxicity and risk of fermented foods, especially meat products (Espinosa-Pesqueira et al., 2018). Additionally, lipid antioxidant enzyme results in the oxidation and acidification of high-fat food products (Mukumbo et al., 2020) and glucose aerogenesis affects the texture of sausages. Moreover, the absence of H_2_S affects the flavor and leads to the spoilage of fermented foods (Luo et al., 2013). Hence, only strains d, e, 2, and 32 were feasible for application in food products, since they didn’t possess the characteristics of amino acid decarboxylase, lipid antioxidant enzyme, glucose aerogenesis, and H_2_S production (Table 2). The strains d and e were further selected as candidate strains owing to their strong color formation effect.

The two strains d and e exhibited a rapid growth rate, which enabled them to become dominant strains and inhibit the growth of other bacteria effectively (Figure 9A). Lucke and Hechelmann reported that, if the initial concentration of sodium chloride in fermented sausages were about 3%, the resultant concentration could go up to more than 3% at the end of the ripening stage (Lucke and Hechelmann, 1987). Our findings show that the tolerance of the two strains to sodium chloride was much stronger than *L. curvatus* and *Pediococcus acidilactici*, the ubiquitous starting cultures used in fermented sausages. In addition, although certain nitrite-replacers have been identified for application in fermented sausages, the addition of sodium nitrite was essential, since no alternative could substitute nitrite completely. Therefore, the starting cultures were required to grow normally in the presence of at least 100 mg/mL of sodium nitrite (Özcelik et al., 2016). Figure 9C shows that the strains grew well in the presence of 120 mg/mL of sodium nitrite. Moreover, the two strains had the ability to rapidly reduce the pH of the fermentation broth (Figure 9D), which is an essential characteristic of starting cultures. Additionally, low pH stimulates the release of actin-derived peptides, which provides unique flavors to the sausages (Berardo et al., 2017). Our results illustrate that the two strains have superior industrial potential for use as starting cultures in meat products than previous reported strains.

## Conclusion

In conclusion, the two LAB strains having both chromogenic and antibacterial activity were screened, and identified as *L. plantarum* using 16S rDNA sequence analysis. They had a higher ability to convert myoglobin and Met-Mb to red Mb-NO than previously reported strains owing to high NOS and Met-Mb reductase activity. Importantly, they had great antibacterial activity through destruction of the biofilm and cytomembrane of indicator bacteria, which has not been confirmed for natural biological colorants by other studies. Besides, the two strains possessed all the good characteristics required for use in meat products, like high growth rate, rapid acid production ability, and strong tolerance to sodium chloride and nitrite. Consequently, the two strains have great potential for application as partial or whole nitrite replacers in meat products, owing to their high potential for synthesizing Mb-No and their antibacterial activity, and the superior dual function for natural biological colorants has not been confirmed by other studies.

## Data Availability Statement

The datasets presented in this study can be found in online repositories. The names of the repository/repositories and accession number(s) can be found in the article/Supplementary Material.

## Author Contributions

YZ was designed the study and drafted the original draft. QY was responsible for the method, data acquisition, curation, and analysis. Both authors contributed to the article and approved the submitted version.

## Conflict of Interest

The authors declare that the research was conducted in the absence of any commercial or financial relationships that could be construed as a potential conflict of interest.

## Footnotes

1 http://blast.ncbi.nlm.nih.gov
