# Supplementary material for: Isolation of Antibacterial, Nitrosylmyoglobin Forming Lactic Acid Bacteria and Their Potential Use in Meat Processing
Source: Front Microbiol. 2020 Jun 19;11:1315. doi: 10.3389/fmicb.2020.01315 (PMC7317118; doi:10.3389/fmicb.2020.01315)
Supplement: Supplementary file 1 [file Data_Sheet_1.pdf]

16SrDNA sequence

Strain d：

GGCGTGCGGTGCTATACATGCAAGTCGAACGAACTCTGGTATTGATTGGTGCTTGCATC
ATGATTTACATTTGAGTGAGTGGCGAACTGGTGAGTAACACGTGGGAAACCTGCCCAG
AAGCGGGGGATAACACCTGGAAACAGATGCTAATACCGCATAACAACTTGGACCGCAT
GGTCCGAGTTTGAAAGATGGCTTCGGCTATCACTTTTGGATGGTCCCGCGGCGTATTAG
CTAGATGGTGGGGTAACGGCTCACCATGGCAATGATACGTAGCCGACCTGAGAGGGTA
ATCGGCCACATTGGGACTGAGACACGGCCCAAACTCCTACGGGAGGCAGCAGTAGGG
AATCTTCCACAATGGACGAAAGTCTGATGGAGCAACGCCGCGTGAGTGAAGAAGGGT
TTCGGCTCGTAAAACTCTGTTGTTAAAGAAGAACATATCTGAAAGTAACTGTTCAGGT
ATTGACGGTATTTAACCAGAAAGCCACGGCTAACTACGTGCCAGCAGCCGCGGTAATA
CGTAGGTGGCAAGCGTTGTCCGGATTTATTGGGCGTAAAGCGAGCGCAGGCGGTTTTT
TAAGTCTGATGTGAAAGCCTTCGGCTCAACCGAAGAAGTGCATCGGAAACTGGGAAA
CTTGAGTGCAGAAGAGGACAGTGGAACTCCATGTGTAGCGGTGAAATGCGTAGATATA
TGGAAGAACACCAGTGGCGAAGGCGGCTGTCTGGTCTGTAACTGACGCTGAGGCTCG
AAAGTATGGGTAGCAAACAGGATTAGATACCCTGGTAGTCCATACCGTAAACGATGAA
TGCTAAGTGTTGGAGGGTTTCCGCCCTTCAGTGCTGCAGCTAACGCATTAAGCATTCC
GCCTGGGGAGTACGGCCGCAAGGCTGAAACTCAAAGGAATTGACGGGGGCCCGCAC
AAGCGGTGGAGCATGTGGTTTAATTCGAAGCTACGC
GAAGAACCTTACCAGGTCTTGACATACTATGCAAATCTAAGAGATTAGACGTTCCCTTC
GGGGACATGGATACAGGTGGTGCATGGTTGTCGTCAGCTCGTGTCGTGAGATGTTGGG
TTAAGTCCCGCAACGAGCGCAACCCTTATTATCAGTTGCCAGCATTAAGTTGGGCACTC
TGGTGAGACTGCCGGTGACAAACCGGAGGAAGGTGGGGATGACGTCAAATCATCATG
CCCCTTATGACCTGGGCTACACACGTGCTACAATGGATGGTACAACGAGTTGCGAACT
CGCGAGAGTAAGCTAATCTCTTAAAGCCATTCTCAGTTCGGATTGTAGGCTGCAACTC
GCCTACATGAAGTCGGAATCGCTAGTAATCGCGGATCAGCATGCCGCGGTGAATACGT
TCCCGGGCCTTGTACACACCGCCCGTCACACCATGAGAGTTTGTAACACCCAAAGTCG
GTGGGGTAACCTTTTAGGAACCAGCCGCCTAAGTGACAGAATT

Stain e：

GGCTGCGGCGTGCTATACATGCAAGTCGAACGAACTCTGGTATTGATTGGTGCTTGCAT
CATGATTTACATTTGAGTGAGTGGCGAACTGGTGAGTAACACGTGGGAAACCTGCCCA
GAAGCGGGGGATAACACCTGGAAACAGATGCTAATACCGCATAACAACTTGGACCGCA
TGGTCCGAGTTTGAAAGATGGCTTCGGCTATCACTTTTGGATGGTCCCGCGGCGTATTA

GCTAGATGGTGGGGTAACGGCTCACCATGGCAATGATACGTAGCCGACCTGAGAGGGT
AATCGGCCACATTGGGACTGAGACACGGCCCAAACTCCTACGGGAGGCAGCAGTAGG
GAATCTTCCACAATGGACGAAAGTCTGATGGAGCAACGCCGCGTGAGTGAAGAAGGG
TTTCGGCTCGTAAAACTCTGTTGTTAAAGAAGAACATATCTGAAAGTAACTGTTCAGG
TATTGACGGTATTTAACCAGAAAGCCACGGCTAACTACGTGCCAGCAGCCGCGGTAAT
ACGTAGGTGGCAAGCGTTGTCCGGATTTATTGGGCGTAAAGCGAGCGCAGGCGGTTTT
TTAAGTCTGATGTGAAAGCCTTCGGCTCAACCGAAGAAGTGCATCGGAAACTGGGAA
ACTTGAGTGCAGAAGAGGACAGTGGAACTCCATGTGTAGCGGTGAAATGCGTAGATAT
ATGGAAGAACACCAGTGGCGAAGGCGGCTGTCTGGTCTGTAACTGACGCTGAGGCTC
GAAAGTATGGGTAGCAAACAGGATTAGATACCCTGGTAGTCCATACCGTAAACGATGA
ATGCTAAGTGTTGGAGGGTTTCCGCCCTTCAGTGCTGCAGCTAACGCATTAAGCATTCC
GCCTGGGGAGTACGGCCGCAAGGCTGAAACTCAAAGGAATTGACGGGGGCCCGCAC
AAGCGGTGGAGCATGTGGTTTAATTCGAAGCTACG
CGAAGAACCTTACCAGGTCTTGACATACTATGCAAATCTAAGAGATTAGACGTTCCCTT
CGGGGACATGGATACAGGTGGTGCATGGTTGTCGTCAGCTCGTGTCGTGAGATGTTGG
GTTAAGTCCCGCAACGAGCGCAACCCTTATTATCAGTTGCCAGCATTAAGTTGGGCAC
TCTGGTGAGACTGCCGGTGACAAACCGGAGGAAGGTGGGGATGACGTCAAATCATCA
TGCCCCTTATGACCTGGGCTACACACGTGCTACAATGGATGGTACAACGAGTTGCGAA
CTCGCGAGAGTAAGCTAATCTCTTAAAGCCATTCTCAGTTCGGATTGTAGGCTGCAACT
CGCCTACATGAAGTCGGAATCGCTAGTAATCGCGGATCAGCATGCCGCGGTGAATACG
TTCCCGGGCCTTGTACACACCGCCCGTCACACCATGAGAGTTTGTAACACCCAAAGTC
GGTGGGGTAACCTTTTAGGAACCAGCCGCCTAAGTGACAGAGTTG
